# Carnitine is a pharmacological allosteric chaperone of the human lysosomal *α*-glucosidase

**DOI:** 10.1080/14756366.2021.1975694

**Published:** 2021-09-27

**Authors:** Roberta Iacono, Nadia Minopoli, Maria Carmina Ferrara, Antonietta Tarallo, Carla Damiano, Caterina Porto, Sandra Strollo, Véronique Roig-Zamboni, Gianfranco Peluso, Gerlind Sulzenbacher, Beatrice Cobucci-Ponzano, Giancarlo Parenti, Marco Moracci

**Affiliations:** a Department of Biology, University of Naples “Federico II”, Complesso Universitario di Monte S. Angelo, Naples, Italy; b Institute of Biosciences and Bioresources – CNR, Naples, Italy; c Telethon Institute of Genetics & Medicine, Pozzuoli, Italy; d Centre National de la Recherche Scientifique (CNRS), Aix-Marseille University, AFMB, Marseille, France; e Research Institute on Terrestrial Ecosystems, UOS Naples-CNR, Naples, Italy; f Department of Translational Medical Sciences, Federico II University, Naples, Italy

**Keywords:** *α*-Glucosidase, glycogen storage disease type 2, orphan drugs, carbohydrate active enzymes, lysosomal disease

## Abstract

Pompe disease is an inherited metabolic disorder due to the deficiency of the lysosomal acid *α*-glucosidase (GAA). The only approved treatment is enzyme replacement therapy with the recombinant enzyme (rhGAA). Further approaches like pharmacological chaperone therapy, based on the stabilising effect induced by small molecules on the target enzyme, could be a promising strategy. However, most known chaperones could be limited by their potential inhibitory effects on patient’s enzymes. Here we report on the discovery of novel chaperones for rhGAA, L- and D-carnitine, and the related compound acetyl-D-carnitine. These drugs stabilise the enzyme at pH and temperature without inhibiting the activity and acted synergistically with active-site directed pharmacological chaperones. Remarkably, they enhanced by 4-fold the acid *α*-glucosidase activity in fibroblasts from three Pompe patients with added rhGAA. This synergistic effect of L-carnitine and rhGAA has the potential to be translated into improved therapeutic efficacy of ERT in Pompe disease.

## Introduction

Glycogen storage disease type 2, or Pompe disease (PD, OMIM 232300) is an inborn metabolic disorder caused by the functional deficiency of the acid lysosomal *α*-glucosidase (GAA, acid maltase, E.C.3.2.1.20), the enzyme hydrolysing *α*-1,4 and *α*-1,6-glucosidic bonds in glycogen and belonging to family GH31 of the carbohydrate-active enzyme (CAZy) classification (www.cazy.org^1^). GAA deficiency results in glycogen accumulation in lysosomes and in secondary cellular damage, with mechanisms not fully understood^2–5^. In PD, muscles are particularly vulnerable to glycogen storage, and disease manifestations are predominantly related to the involvement of cardiac and skeletal muscles. However, central nervous system involvement is emerging as part of the clinical spectrum in infantile-onset patients^6^.

It is assumed that to obtain positive therapeutic effects it is enough that the enzymatic activity of GAA is rescued at about 10% of the wild type, meaning that a relatively small increase in activity can mitigate the clinical course^2^. Therapeutic strategies include the supply of wild type enzymes, such as enzyme replacement therapy (ERT), gene therapy, or small-molecule drugs able to adjust cellular networks controlling protein synthesis, folding, trafficking, aggregation, and degradation, thus facilitating the escape of mutated proteins from the endoplasmic reticulum-associated degradation (ERAD) machinery^7–10^.

Since 2006, enzyme replacement therapy (ERT) with recombinant human *α*-glucosidase has been approved and is currently considered the standard of care for the treatment of PD, improving survival by stabilising the disease course^6^^,^^11–13^. However, limitations are also known, in fact, despite treatment, some patients experience little clinical benefit or show signs of disease progression^14^. Several factors concur in limiting the therapeutic success of ERT, including the age at the start of treatment^15^^,^^16^, the immunological status of patients^17^, the insufficient targeting of the enzyme to skeletal muscle^18^, the possible instability at neutral pH of the recombinant enzyme during the transit to lysosomes^19–21^, the relative deficiency of the cation-independent mannose-6-phosphate receptor, required for enzyme uptake in muscle cells^22^^,^^23^, and the build-up of the autophagic compartment observed in myocytes^24–26^.

For all the reasons pointed above, alternative treatments, like pharmacological chaperone therapy (PCT), would be highly desirable. This approach, which has been designed for the treatment of protein misfolding diseases (PMD), exploits small-molecule ligands that may bind directly to the defective enzymes, templating the folding of proteins in the most stable conformation(s) and preventing their recognition and disposal by the ERAD machinery^27–30^.

**Figure 1.**
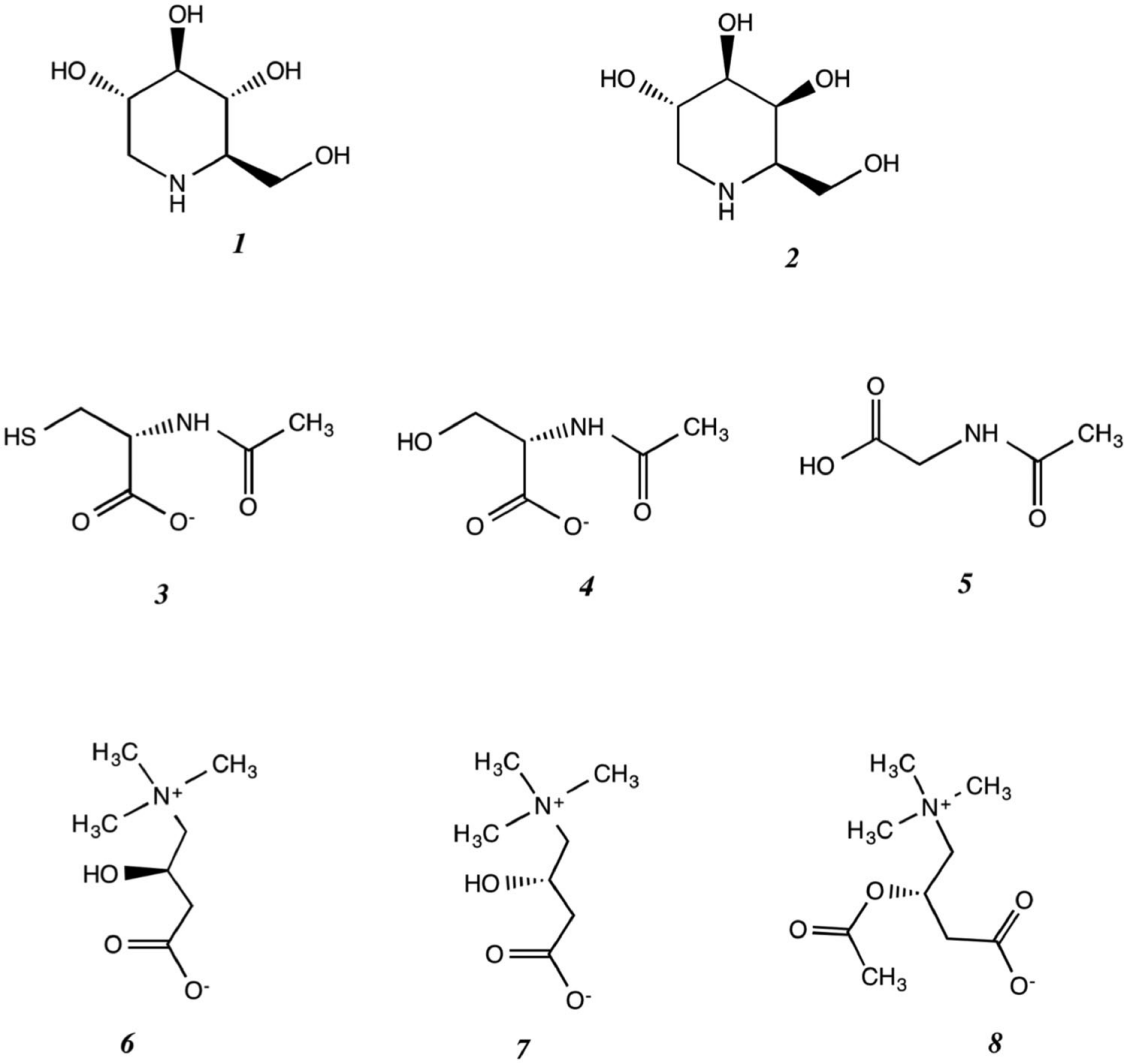
Pharmacological chaperones for lysosomal storage diseases. deoxynojirimycin (DNJ) (***1***), 1-deoxy-galactonojirimycin (DGJ) (***2***), *N*-acetylcysteine (NAC) (***3***), *N*-acetylserine (NAS) (***4***), *N*-acetylglycine (NAG) (***5***), L-carnitine (L-CAR) (***6***), D-carnitine (D-CAR) (***7***), and acetyl-carnitine (A-D-CAR) (***8***).

Most pharmacological chaperones (PC) proposed or used for the treatment of lysosomal storage diseases (LSD) are reversible competitive inhibitors of the target enzymes. Compared to ERT, small-molecule chaperones have important advantages in terms of biodistribution, oral availability, and reduced impact on patients’ quality of life. Recent studies have shown that 1-deoxynojirimycin, *N*-butyl-deoxynojirimycin (DNJ, ***1*** and NB-DNJ, respectively, Figure 1), and 1-deoxygalactonojirimycin (DGJ, ***2***), may also potentiate the effects of the enzymes used for ERT in Pompe^31^ and Fabry diseases, respectively^21^^,^^32^. However, these active-site-directed PCs interfere with the activity of the targeted enzymes^5^^,^^33^. The paradox that an inhibitor can increase the enzymatic activity is explained by the fact that therapeutic levels can be reached at sub-inhibitory intracellular concentrations and that the high concentrations of the natural substrate accumulated in the lysosome or the acidic conditions within the organelle may displace the PC inhibitor from the active site.

An ideal chaperone should be able to protect the enzymes from degradation without interfering with its activity, be largely bioavailable in tissues and organs, reach therapeutic levels in cellular compartments where its action is required, show high specificity for the target enzyme with negligible effects on other enzymes, and have a good safety profile. The extensive search for new PCs is currently being performed by high-throughput screening of chemical libraries^34^ for molecules specific for GAA^35^, *β*-glucoceramidase (GCase)^36–38^, and *β*-hexosaminidase^39^, or by biochemical characterisation of known inhibitors^40^. However, a reason of major concern on the clinical use is that the majority of PCs identified so far for the treatment of LSD are active-site directed competitive inhibitors^33^.

We focussed our search on drugs already approved for human therapy for their rapid clinical translation without the need for phase I clinical trials. We found that *N*-acetylcysteine (NAC, ***3***, Figure 1), a known pharmaceutical drug, and the related aminoacids *N*-acetylserine (NAS, ***4***) and *N*-acetylglycine (NAG, ***5***), structurally unrelated to known inhibitors of GAA, behave like novel allosteric PCs for this enzyme^41^. These molecules stabilise rhGAA at non-acidic pH, enhanced the residual activity of mutated GAA, and improved the efficacy of rhGAA used for ERT in Pompe disease^41^^,^^42^. The high-resolution 3 D-structure of rhGAA in complex with NAC allowed to identify two binding sites for this PC in regions distant from the active site, and to explain the chaperoning activity of NAC^43^.

Following the same approach, here we report on the results of screening for other putative allosteric chaperones, already approved as drugs or nutraceuticals. We found that L-carnitine (L-CAR, ***6*** in Figure 1), D-carnitine (D-CAR, ***7***), and the related compound acetyl-D-carnitine (A-D-CAR, ***8***) can stabilise rhGAA at non-lysosomal pH and improve the activity of GAA in PD patient's fibroblasts. Therefore, these molecules are novel potential pharmacological chaperones with excellent perspectives for the treatment of Pompe disease alone and in combination with ERT.

## Materials and methods

### Reagents

rhGAA (*α*-glucosidase, Myozyme), was from Genzyme Co, Cambridge, MA, USA. As a source of enzyme, authors used the residual amounts of the reconstituted recombinant enzyme prepared for the treatment of PD patients at the Department of Translational Medical Sciences of the University of Naples, “Federico II”. D-CAR, A-D-CAR were from Sigma-tau; L-CAR, DNJ, and 4NP-Glc were purchased from Sigma–Aldrich.

### Enzyme characterisation

The standard activity assay of rhGAA was performed in 200 µL by using 0.2 µM at 37 °C in 100 mM sodium acetate pH 4.0 and 20 mM 4NP-Glc. The reaction was started by adding the enzyme. After 2 min incubation time the reaction was blocked by adding 800 µL of 1 M sodium carbonate pH 10.2. Absorbance was measured at 420 nm at room temperature and an extinction coefficient of 17.2 mM^−1 ^cm^−1^ was used to calculate enzymatic units. One enzymatic unit is defined as the amount of enzyme catalysing the conversion of 1 μmol substrate into the product in 1 min, under the indicated conditions.

The effect of pH on the rhGAA stability was measured by preparing reaction mixtures containing 6.8 µM of enzyme in the presence of 50 mM sodium phosphate, pH 7.4. After incubations at 37 °C, aliquots were withdrawn at the times indicated in Results and the residual *α*-glucosidase activity was measured with the standard activity assay described above. To test the effect on the pH stability of rhGAA by the chaperons, experiments were performed as described above by adding to the reaction mixtures the amounts of the different compounds indicated in the Results.

### Thermal stability of rhGAA

Thermal stability experiments of rhGAA were performed as described in Porto et al.^41^ and the dissociation constant of L-CAR was measured as described in Roig-Zamboni et al.^43^. Briefly, 0.9 µM of the enzyme were incubated in the absence and in the presence of L-CAR, D-CAR A-D-CAR, NAC, and DNJ in 25 mM sodium phosphate buffer, pH 7.4, and 150 mM NaCl.

The effect of L-CAR on rhGAA stability was tested by analysing the specific activity. L-CAR at various concentrations was incubated with rhGAA and the enzymatic specific activity was measured after 5 h of incubation at pH 7.4.

Thermal stability scans were performed at 1 °C/min in the range 25–95 °C in a Real-Time LightCycler (Bio-Rad). Differential Scanning Fluorimetry (DSF) scans were performed at ten concentrations of L-CAR (from 2 to 20 mM) and changes in the fluorescence of SYPRO Orange dye were monitored as a function of temperature at pH 7.4. Thermal scans were performed in triplicate and melting temperatures were calculated according to Niesen et al.^44^. For the determination of the dissociation constant (*K_D_*) of L-CAR experimental data were best fitted according to a simple cooperative model equation reported in Vivoli et al.^45^ by using the software GraphPAD Prism (GraphPad Software, San Diego, CA, USA). The melting temperature values were plotted as a function of ligand concentration.

### Fibroblast cultures

Fibroblasts from PD patients were derived from skin biopsies after obtaining the informed consent of patients. Normal age-matched control fibroblasts were available in the laboratory of the Department of Paediatrics, Federico II University of Naples. All cell lines were grown at 37 °C with 5% CO_2_ in Dulbecco’s modified Eagle’s medium (Invitrogen, Grand Island, NY, USA) and 20% foetal bovine serum (Sigma–Aldrich, St Louis, MO, USA), supplemented with 2 mM/L glutamine, 100 U/ml penicillin and 100 µg/ml streptomycin.

### Incubation of fibroblasts with rhGAA and GAA assay

To study the rhGAA uptake and correction of GAA activity in PD fibroblasts, the cells were incubated with 50 µM rhGAA for 24 h, in the absence or in the presence of 10 mM L-CAR. Untreated cells were used for comparison. After the incubation, the cells were harvested by trypsinization and disrupted by 5 cycles of freezing and thawing.

GAA activity was assayed by using the fluorogenic substrate 4-methylumbelliferyl-*α*-D-glucopyranoside (4MU) (Sigma–Aldrich) according to a published procedure^31^. Briefly, 25 µg of cell homogenates were incubated with the fluorogenic substrate (2 mM) in 0.2 M acetate buffer, pH 4.0, for 60 min in incubation mixtures of 100 µl. The reaction was stopped by adding 1 ml of glycine-carbonate buffer, 0.5 M, pH 10.7. Fluorescence was read at 365 nm (excitation) and 450 nm (emission) on a Promega GloMax Multidetection system fluorometer. Protein concentration in cell homogenates was measured by the Lowry assay.

### Immunofluorescence analysis and confocal microscopy

For immunofluorescence studies, cells (human fibroblasts) grown on coverslips were fixed using methanol (5 min at −20 °C to study the colocalization GAA-LAMP2), permeabilized using 1% PBS (phosphate-buffered saline)—Triton 0,1% and blocked with 0.05% saponin, 1% BSA diluted in 1% PBS (blocking solution) at room temperature for 1 h. The cells were incubated with the primary antibodies anti-GAA rabbit polyclonal antibody (PRIMM) and anti-LAMP2 mouse monoclonal antibody (Santa Cruz Biotechnology), and diluted in blocking solution overnight at 4 °C. Then, cells were washed with 1% PBS and then incubated with appropriate autofluorescent secondary antibodies (anti-rabbit or anti-mouse antibodies conjugated to Alexa Fluor 488 or 596) and DAPI (4′,6-diamidino-2-phenylindole, Invitrogen) in 0.05% saponin, 3% BSA, 1% PBS. Samples were then washed, mounted with Mowiol (Sigma), and examined with a Zeiss LSM700 confocal microscope. Colocalization and quantitative analysis were performed with Fiji (ImageJ, NIH, USA) software.

## Results

### L-CAR improves rhGAA stability in vitro

In the framework of our search for novel PCs, which led to the identification of NAC/NAS/NAG (***3–5*** in Figure 1)^41^, we embarked on the search of molecules already approved as pharmaceutical drugs and/or nutraceuticals that can be rapidly introduced in therapeutic treatments without the need of long and expensive clinical trials. Among the molecules considered, L-carnitine (***6***) was identified as a possible target. In addition, D-carnitine and the related compound acetyl-D-carnitine were also analysed. L-CAR is a well-known conditionally essential micronutrient and nutraceutical^46^^,^^47^, whereas for D-CAR side effects have been documented, including toxicity in patients treated with dialysis, and in rats and fishes^48–50^. In fact, most of the study was performed on L-CAR.

To test this molecule on GAA, we analysed its effect on the pH stability of the enzyme similarly to previous studies on lysosomal enzymes^20^^,^^41^^,^^51^. In particular, we analysed rhGAA residual activity on 100 mM 4-nitrophenyl-*α*-D-glucopyranoside (4NP-Glc) in 100 mM sodium acetate buffer, pH 4.0. These assays, in which rhGAA is optimally active and stable for up to 24 h, were used to test the enzyme stability. In fact, at acidic or neutral pHs (pH 3.0 and 7.0, respectively), which are lower and higher, respectively, to the one of the lysosomal compartments, the enzyme halved its activity in about 5 h^41^.

**Figure 2.**
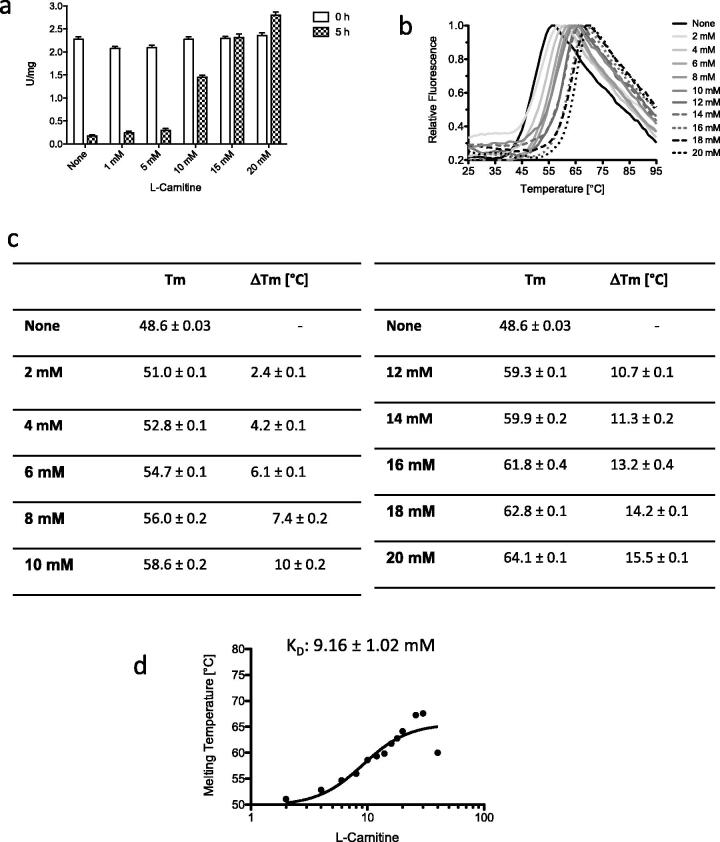
Comparison of the effect of L-carnitine on the stability of rhGAA. (a) Effect of L-CAR on the rhGAA stability; (b) Effect of L-CAR on the structural stability of rhGAA; (c) Summary of the *T_m_s* measured by DSF; (d) Determination of the K_D_ rhGAA-L-CAR by DSF.

L-CAR, already at the concentration of 10 mM, rescued the activity of rhGAA on 4NP-Glc after 5 h of incubation at pH 7.4 (Figure 2(a)). The stabilising effect on the rhGAA activity was maintained even after 48 h of incubation in the presence of 20 mM L-CAR (Figure S1(a)). No effect on the specific activity of rhGAA was observed when L-CAR at any concentration was included in the *α*-glucosidase assay, indicating that it did not interact with the active site of the enzyme (Figure S1(b)).

Interestingly, L-CAR increased in a dose-dependent manner also the structural stability of rhGAA as analysed by DSF (Figure 2(b)). The variations of the melting temperature (Δ*T_m_*) increased by about 2 °C at every 2 mM increment of L-CAR concentration (Figure 2(c)).

The dissociation constant of L-CAR for rhGAA was measured by DSF according to Vivoli et al.^45^ (Figure 2(d)). L-CAR showed a *K_D_* similar to that of the allosteric chaperone NAC (9.16 ± 1.02 and 11.57 ± 0.74 mM, respectively)^43^. As expected for molecules that do not bind to the rhGAA active site, these values are higher than the typical *K_i_* of 3.4 µM exhibited by active-site directed molecular chaperones, such as the DNJ inhibitor^41^.

### Effect on rhGAA stability by the combined action of allosteric and active-site directed PCs

Similar stabilising effects were also observed with the related compounds D-CAR and A-D-CAR (Figure 1, ***7*** and ***8***, respectively). Both compounds rescued the activity of rhGAA on 4NP-Glc after 5 h of incubation at pH 7.4 (Figure S2(a)). Again, no effect on the specific activity of rhGAA at 0.1–10 mM concentrations was observed (Figure S2(b)), indicating that D-CAR and A-D-CAR also did not interact with the active site of the enzyme. Compared to the L-isomer (compare ***6*** and ***7*** in Figure 1), D-CAR showed a complete rescue of rhGAA activity already at 10 mM concentration *vs.* 20 mM of L-CAR (Figure S2(c)), maintaining the stabilising effect even after 24 h of incubation (Figure S1(a)). DSF analysis showed that D-CAR increased the structural stability of rhGAA in a dose-dependent manner (Figure S2(d)) and that the Δ*T_m_* increased by about 2 °C at every 2 mM increment of D-CAR concentration (Figure S2(e)).

**Figure 3.**
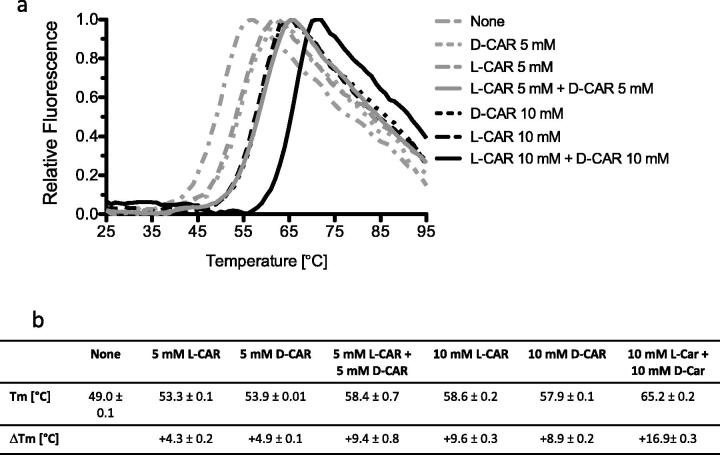
Effect of a racemic mixture of D/L-CAR on the structural stability of rhGAA. (a) DSF analysis. L-CAR and D-CAR were incubated with rhGAA either alone (5 and 10 mM) or in combination (at 5 or 10 mM each). (b) Summary of the T_m_ measured by DSF.

To test if carnitine L- and D-enantiomers had additive effects, we analysed rhGAA stability in the presence of equimolar amounts of D- and L-CAR. As shown in Figure 3, when rhGAA was incubated with 10 mM total concentration of the two enantiomers (resulting from L-CAR 5 mM + D-CAR 5 mM), the Δ*T_m_s* of 9.4 ± 0.8 °C corresponds to the sum of the Δ*T_m_s* measured when the enzyme was incubated with either L- or D-CAR at 5 mM concentration (Δ*T_m_s* of 4.3 ± 0.2 and 4.9 ± 0.1 °C, respectively). A similar additive effect was observed when the concentration of each enantiomer was increased to 10 mM of D- and L-CAR (Figure 3(b)).

**Figure 4.**
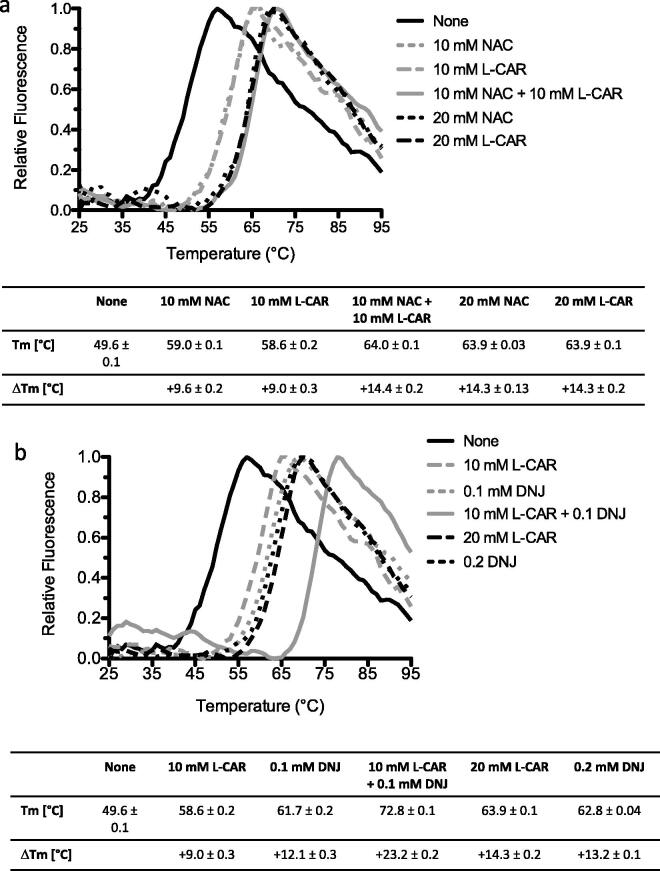
Comparison of the effect of allosteric and non-allosteric chaperones on the stability of rhGAA. (a) Analysis of the synergistic effect of L-CAR and NAC. rhGAA was incubated with L-CAR either alone (10 or 20 mM) or in combination with NAC, at 10 mM each. (b) Analysis of the synergistic effect of L-CAR and DNJ. L-CAR was incubated with rhGAA either alone (10 or 20 mM) or in combination with DNJ (0.1 mM).

The combined effect on rhGAA by L-CAR and other active-site directed or allosteric chaperones is shown in Figure 4. At a concentration of 10 mM, L-CAR increased the T_m_ of rhGAA by 9.0 ± 0.3 (T_m_ 58.6 ± 0.2 *vs.* 49.6 ± 0.1 °C of rhGAA alone) a value similar to that obtained with NAC at the same concentration (9.6 ± 0.2 °C), but slightly lower than that of the active-site directed pharmacological chaperone DNJ (Figure 1: ***1***) at a concentration of 0.1 mM (+12.1 ± 0.3 °C) (Figures 4(a,b)). To understand the mechanism of stabilisation towards rhGAA we combined these molecules in DSF experiments. L-CAR was mixed at 10 mM concentration in equimolar ratios with NAC (Figure 4(a)) or with 0.1 mM DNJ (Figure 4(b)). The stabilising effect of L-CAR in the presence of 10 mM equimolar amounts of NAC (20 mM total) was identical to the effect observed when each of the allosteric PCs was used individually at 20 mM concentration (Figure 4(a)). However, when L-CAR and NAC were combined, the ΔT_m_ of 14.4 ± 0.2 °C was almost identical to those observed when L-CAR and NAC were used singularly at 20 mM concentration each (14.3 ± 0.2 and 14.3 ± 0.1 °C, respectively) (Figure 4(a)).

The Δ*T_m_s* obtained with either 10 mM L-CAR combined with 0.1 mM of the active-site directed allosteric chaperone DNJ, were exactly additive with ΔT_m_ of +9.0 ± 0.3, +12.1 ± 0.3, and +23.2 ± 0.2 °C with L-CAR, DNJ, and L-CAR + DNJ, respectively, confirming that these PCs interact with different sites of rhGAA (Figure 4(b)).

### Effect of L-CAR in PD fibroblasts

**Figure 5.**
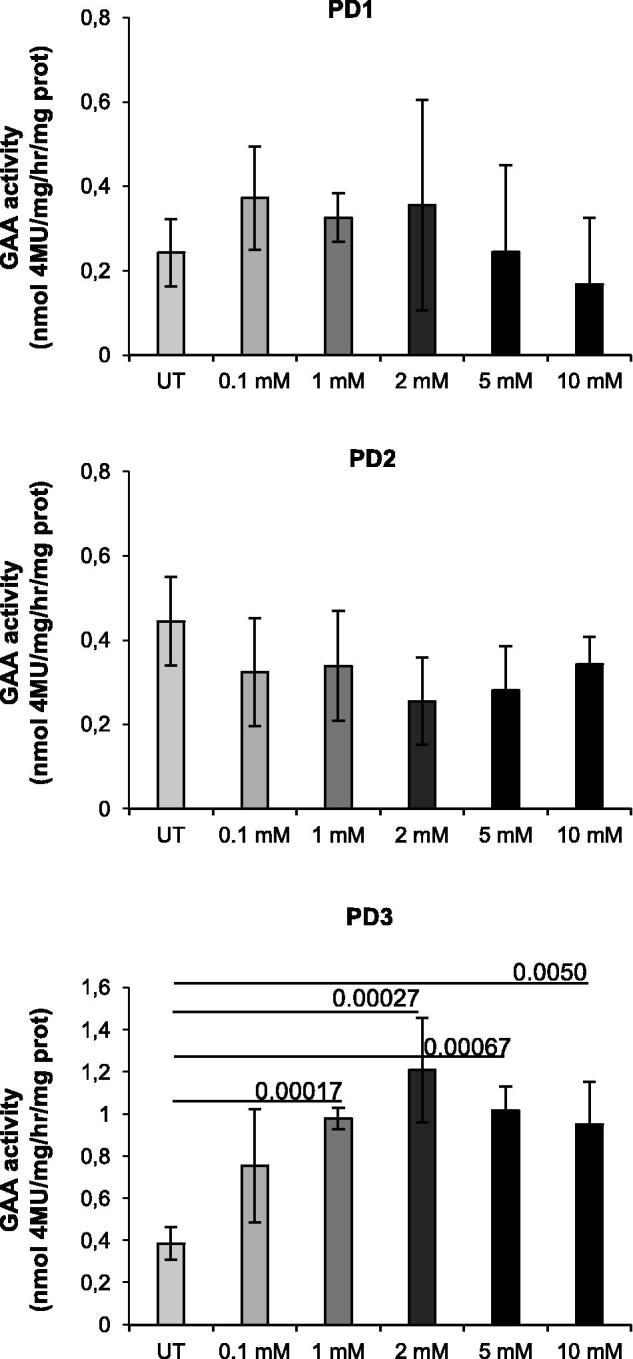
Effect of L-CAR in PD fibroblasts. (a) Effect of L-CAR on the residual activity of mutated GAA in fibroblasts. Fibroblasts derived from three PD patients were incubated in the presence and in the absence of 0.1–10 mM L-CAR before being harvested and used for GAA assay. The untreated cells (UT) were used as a control. The chaperone has significant effects on endogenous residual activity in the cells from patient 3.

We studied the effect of L-CAR on mutant GAA activity in cultured fibroblasts from three PD patients carrying different mutations and with early-onset phenotypes (see Table 1). Fibroblasts were incubated in the presence of 0.1 to 10 mM L-CAR for 24 h and the GAA activity was compared to that obtained in untreated cells. The chaperone had negligible and non-significant effects on endogenous residual activity in the cells from patients 1 and 2, while significantly enhancing effects were seen in cells from patient 3, homozygous for the p.L552P mutation, that had been already reported to be responsive to the active site-directed chaperones DNJ and NB-DNJ (Figure 5)^52^. Significant increments in activity were observed in a range of L-CAR concentrations between 1 and 10 mM, with a 2.8-fold increase at 2 mM.

**Table 1.** Mutants used in this study.

Patient ID	Mutation allele 1	Mutation allele 2	Phenotype
PD 1	p.L552P	p.P79Rfs*12	Infantile-onset classic
PD 2	p.R375L	p.V755Sfs*41	Infantile-onset classic
PD 3	p.L552P	p.L552P	Infantile-onset atypical

It has been shown previously that active site-directed chaperones enhance the activity of recombinant enzymes used for ERT in PD and Fabry disease^31^^,^^32^, with a synergistic effect. In PD fibroblasts the iminosugar NB-DNJ enhanced rhGAA efficacy by ∼1.3- to 2-fold. An enhancing effect on correction of GAA deficiency by rhGAA and a better enzyme processing was also demonstrated with the allosteric chaperone NAC^41^.

**Figure 6.**
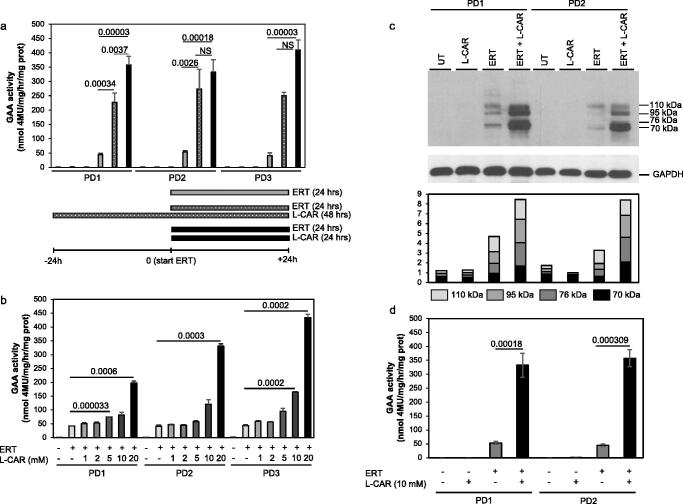
Synergy between L-CAR and rhGAA in PD fibroblasts. (a) Setting the conditions for evaluation of synergy between L-CAR and rhGAA. Different treatment protocols were evaluated: (i) pre-incubation of cells with L-CAR for 24 h, followed by co-incubation of L-CAR and rhGAA for an additional 24 h; (ii) co-incubation of L-CAR and rhGAA for 24 h. (b) Setting the optimal L-CAR concentrations for evaluation of synergy between L-CAR and rhGAA. Fibroblasts were incubated with rhGAA and different L-CAR concentrations (1–20 mM). GAA activity enhancements were observed at 5, 10, and 20 mM L-CAR concentrations with the highest and statistically most significant enhancements at 10 and 20 mM. (c) Effect of L-CAR on rhGAA processing in PD fibroblasts. Cells were incubated for 24 h with rhGAA alone or with rhGAA in combination with 10 mM L-CAR. In the cells treated with the combination of rhGAA and L-CAR the amount of the 70–76 kDa mature GAA active peptides were dramatically improved, as indicated by quantitative analysis by western blot. Glyceraldehyde 3-phosphate dehydrogenase (GAPDH) is the loading control. (d) GAA activities measured in PD fibroblasts. The increase of GAA activity confirms the enhancing effect of L-CAR.

We tested whether the allosteric PC L-CAR also shows a similar effect in combination with ERT in the three cell lines indicated above. We first studied the optimal conditions to evaluate this effect. We compared a protocol based on pre-incubation of cells with L-CAR for 24 h, followed by co-incubation of L-CAR and rhGAA for an additional 24 h, with a protocol based on co-incubation of L-CAR and rhGAA for 24 h (Figure 6(a)). The results of both protocols were compared with those obtained in cells treated with rhGAA alone. The second treatment protocol gave the best results and was selected to evaluate the optimal L-CAR concentration for rhGAA enhancement.

With the co-dosing of rhGAA and L-CAR (1–20 mM) GAA activity enhancements were observed at 5, 10, and 20 mM L-CAR concentrations (Figure 6(b)). The highest and statistically most significant enhancements were obtained at 10 and 20 mM. Higher L-CAR concentrations (up to 50 mM) were toxic for fibroblasts (not shown). Thus, we selected the concentration of 10 mM for further experiments, as this concentration appeared to combine efficacy and safety for cells.

We next studied the effect of L-CAR on rhGAA processing in PD1 and PD2 fibroblasts. For enzyme replacement therapy rhGAA is provided by the manufacturer as a 110 kDa precursor. Once internalised by cells through the mannose-6-phosphate receptor and the endocytic pathways, the enzyme is converted into an intermediate of 95 kDa and the active molecular proteoforms of 76 and 70 kDa. Cells were incubated for 24 h with rhGAA alone or with rhGAA in combination with 10 mM L-CAR. In the cells treated with the combination of rhGAA and L-CAR the amount of the 70–76 kDa mature GAA active peptides was dramatically improved (Figure 6(c)). The corresponding GAA activities measured in PD1 and PD2 cells (Figure 6(d)) confirmed the enhancing effect of L-CAR and were in line with those observed in previous experiments.

**Figure 7.**
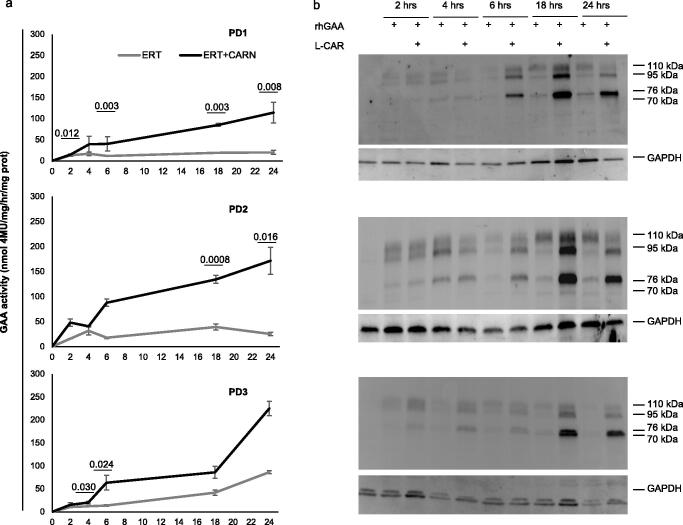
Kinetics of GAA enhancements at different time-points in PD fibroblasts treated with rhGAA alone or in combination with 10 mM L-CAR. (a) GAA activity increased progressively over time and an enhancing effect of co-incubation with L-CAR was already detectable at 2 h and became progressively more pronounced up to 24 h (a). The amounts and the processing of rhGAA, analysed by western blot, also improved over time (b).

We also studied the kinetics of GAA enhancements at different time points in PD fibroblasts treated with rhGAA alone or in combination with 10 mM L-CAR. GAA activity increased progressively over time and an enhancing effect of co-incubation with L-CAR was already detectable at 2 h and became progressively more pronounced up to 24 h (Figure 7(a)). The amounts and the processing of rhGAA, analysed by western blot, also improved over time (Figure 7(b)).

**Figure 8.**
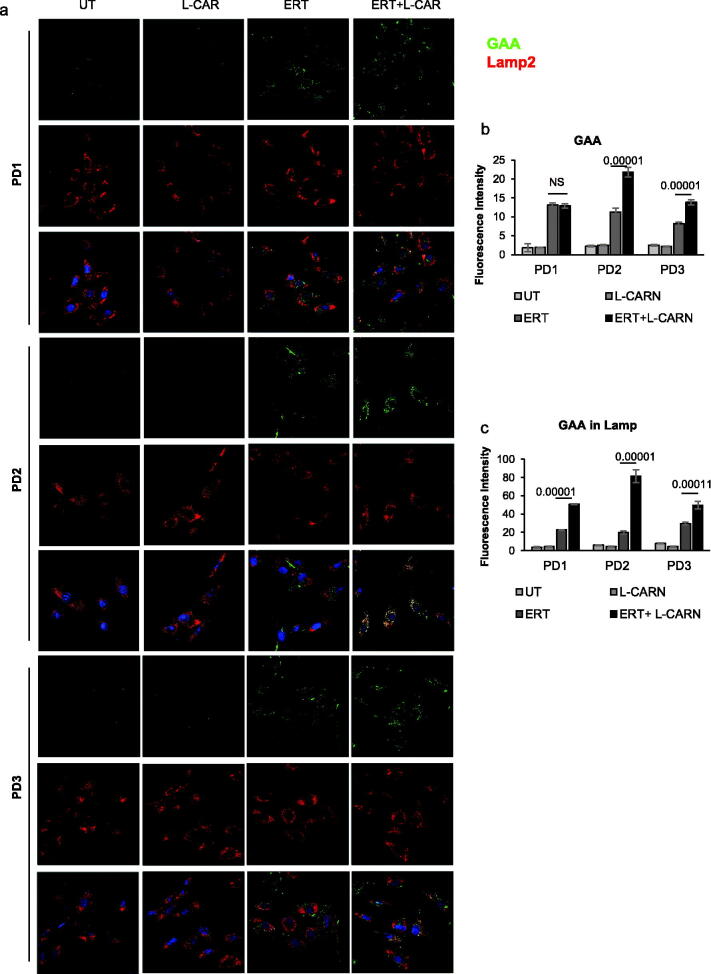
Effects of rhGAA and L-CAR co-dosing on lysosomal trafficking of the recombinant enzyme. The cells of three Pompe patients were incubated under the conditions selected in the previous experiments, and co-localization of rhGAA with Lamp2 was analysed by confocal immune-fluorescence microscopy. In all three cells lines the co-localization was improved (a). This result was confirmed by a quantitative analysis of total GAA signal (b) and of GAA signal co-localized with Lamp2 (c).

We next looked at the effects of rhGAA and L-CAR co-dosing on the lysosomal trafficking of the recombinant enzyme. The cells were incubated under the conditions selected in the previous experiments, and co-localization of rhGAA with the lysosomal associated membrane protein 2 (Lamp2), a common lysosomal tag, was analysed by confocal immune-fluorescence microscopy. In all three cells lines the co-localization was improved (Figure 8(a)). This result was confirmed by a quantitative analysis of the total GAA signal (Figure 8(b)) and of the GAA signal co-localized with Lamp2 (Figure 8(c)) performed by ImageJ Software.

## Discussion

The problems connected to the inhibitory effect of active-site-directed PCs currently used in clinics for LSD can be addressed by the identification of novel allosteric chaperones, that, not binding to the active site of the enzyme, are non-inhibitory and can be potentially more effective than active-site directed PCs. Several studies demonstrated that this can be a convenient avenue for the treatment of Gaucher^53^^,^^54^ and Pompe diseases^41^^,^^55^. Another limitation of inhibitors acting as PCs is that they are effective in rescuing only some disease-causing missense mutations, mainly located in the catalytic environment of enzyme scaffolds, and are thus potentially effective only in a limited number of patients. For PD, it was proposed that about only 10–15% of patients may be amenable to PCT with the iminosugar DNJ^56^.

Based on our previous experience with NAC, and related compounds NAS and NAG^41^, we embarked on the identification of novel allosteric pharmacological chaperons for PD. In the study presented here, we show that L-CAR and the related compounds D-CAR and A-D-CAR can stabilise GAA without interfering with its activity. In cell-free assays, these PCs prevented the loss of GAA activity at pH 7.0 and increased the enzyme thermal stability in a concentration-dependent manner like previously shown with NAC^41^^,^^43^. In addition, the combination of L-CAR and the active site-directed chaperone DNJ showed clearly an additive effect (Figure 4(b)) confirming that the two molecules bind to different sites of the enzyme.

The crucial experiment demonstrating the efficacy of L-CAR on PD was the correction of the enzyme defect in patient’s fibroblasts to a greater extent than that observed with NB-DNJ. When the recombinant enzyme was administered to the patient's fibroblasts in combination with L-CAR, the lysosomal trafficking, the maturation, and the intracellular activity of the enzymes increased up to 4-fold when compared to the combination ERT/NB-DNJ treatment (Figures 6–8)^31^.

The ability of L-CAR and its derivatives to bind to rhGAA is rather surprising and previously unpredictable since L-CAR is structurally different from the DNJ and NAC (Figure 1). The main difference between L-CAR/NAC on one hand and DNJ/NB-DNJ on the other on stabilising rhGAA is the 2-fold higher concentration used for the formers to observe similar Δ*T_m_s*. The µM affinity of active-site directed iminosugars, as deduced from *K_i_* values, can be explained by the structural similarity with GAA natural substrates. On the other hand, the mM values of the *K_D_* of the allosteric pharmacological chaperones indicate lower affinity but a specificity higher than those of chemical chaperones or compatible solutes (e.g. osmolytes, sugars, amino acids, etc.), working at molar concentrations^57^^,^^58^.

We demonstrated by the inspection of the high-resolution 3 D-structure of the rhGAA/NAC complex, that NAC could bind at the interface between the catalytic and auxiliary domains, thereby explaining its chaperoning activity by enhancing the structural stability of the overall enzyme’s scaffold and by preventing deleterious oxidation of Cys938^43^. The binding mode of L-CAR is currently not known. The sigmoidal saturation curve for rhGAA when incubated with the L-CAR indicated cooperativity in the binding mode of this allosteric chaperone (Figure 2(d)). In addition, the non-additive stabilising effect of L-CAR when used with NAC (Figure 4) and their similar *K_D_*, suggesting that these molecules could bind to rhGAA by a similar mechanism. The carboxylate group of NAC makes water-mediated contacts in two different sites of rhGAA^43^. Possibly, also carnitine derivatives might form weak interactions with the enzyme, through their carboxylates groups like the N-acetylated amino acids (***3***–***8*** in Figure 1). Structural data that would be needed to understand the mechanism of action of D- and L-CAR are complicated by the weak binding of these molecules to rhGAA. We endeavoured manifold trials to obtain crystal structures of rhGAA in complex with both L-CAR or D-CAR, employing respectively crystal-soaking and co-crystallisation techniques, but unfortunately, our efforts were not coronated by success. The reasons of these failures might be attributed either to the weak binding of the chaperones to rhGAA (although massive doses had been used) or to the fact that the genuine binding sites were obstructed by molecular packing arrangements within the crystal lattice. In this context it is noteworthy mentioning that crystallisation conditions for rhGAA are extremely stringent, with all the rhGAA structures reported in the Protein Data Bank having been obtained (by us or by others) in exactly the same conditions, explaining why experimental settings are not favourable to unveil binding-sites hidden by crystal-lattice contacts.

The presence in proteins of weak binding sites for small molecules has been predicted and several experimental and *in silico* studies showed “hotspots” on protein surfaces that can bind weakly to small molecules, even at low M range, expanding potential druggable sites^59–62^. Thus, further studies are needed to identify carnitine binding sites on rhGAA, however, our study suggests that other molecules, whose chaperoning activity cannot be simply inferred from their molecular structure, may be effective as PCs for LSDs, thereby opening new and wider opportunities for the identification of novel therapeutic drugs.

The use of L-carnitine as a drug for the treatment of PD is particularly attractive. L-CAR is involved in fatty acid metabolism and synthesised mainly in the liver and kidneys from the essential amino acids lysine and methionine as ultimate precursors to form trimethyl lysine. L-CAR is not toxic at the concentration normally administrated and its use is approved as nutraceutical. Instead, the use of D-carnitine and acetyl-D-carnitine in clinics is less reliable. D-Carnitine can interfere with the uptake and transport of L-carnitine by inhibiting the carnitine acetyltransferase and its use in patients affected by kidney illnesses is avoided^49^. In addition, documented clinical use of acetyl-D-carnitine and its pharmacologically acceptable salts is limited to the therapeutic treatment of glaucoma^63^. Therefore, L-CAR might be promptly included in clinical protocols for the treatment of PD while its D-CAR and A-D-CAR derivatives need more investigations.

The enzyme/PCs molar ratios used to obtain the stabilisation of rhGAA described here, ranged from 1:10^2^ to 1:10^4^ for DNJ and L-CAR/NAC, respectively. This indicates that even the more specific inhibitor DNJ is used at saturating conditions and that L-CAR, showing a lower affinity for the allosteric binding sites on rhGAA, required relatively high concentrations to promote a stabilising effect. However, the toxicity of L-CAR is reported to be low even at doses higher than those used in our study. In fact, L-CAR, with doses of 3 g daily as an oral supplement, is used to treat patients affected by congestive heart failure, end-stage renal disease, hyperthyroidism, male infertility, myocarditis, polycystic ovary syndrome, and toxic side effects caused by the drug valproic acid. Instead, an intravenous infusion of 60 mg/kg of L-CAR is used for patients suffering from angina pectoris^64^.

The synergy between L-Car and ERT demonstrated here may be translated into improved clinical efficacy of ERT, as proposed for other PCs in Gaucher, Pompe, and Fabry diseases^20^^,^^21^^,^^31^^,^^32^. It is worth noting that, while the activity enhancement of endogenous defective enzymes by chaperones in most cases resulted in minor changes in terms of residual activity, likely leading to a modest impact on patients’ outcomes, the synergy between ERT and L-CAR based PCT has the potential to determine remarkable increases of specific activity, independently of mutations affecting individual patients.

## Supplementary Material

Supplemental Material

## References

[1] Lombard V, Golaconda Ramulu H, Drula E, et al. The carbohydrate-active enzymes database (CAZy) in 2013. Nucleic Acids Res 2014;42:D490–5. 24270786 10.1093/nar/gkt1178 PMC3965031

[2] van der Ploeg AT, Reuser AJ. Pompe's disease. Lancet 2008;372:1342–53. 18929906 10.1016/S0140-6736(08)61555-X

[3] Shea L, Raben N. Autophagy in skeletal muscle: implications for Pompe disease. Int J Clin Pharmacol Ther 2009;47 Suppl 1:S42–S47. 20040311 10.5414/cpp47042 PMC2948975

[4] Parenti G, Andria G. Pompe disease: from new views on pathophysiology to innovative therapeutic strategies. Curr Pharma Biotechnol 2011;12:902–15. 10.2174/138920111795542606 21235442

[5] Parenti G, Moracci M, Fecarotta S, Andria G. Pharmacological chaperone therapy for lysosomal storage diseases. Future Med Chem 2014;6:1031–45. 25068986 10.4155/fmc.14.40

[6] McIntosh PT, Hobson-Webb LD, Kazi ZB, et al. Neuroimaging findings in infantile Pompe patients treated with enzyme replacement therapy. Mol Genet Metab 2018;123:85–91. 29050825 10.1016/j.ymgme.2017.10.005 PMC5808895

[7] Mu TW, Ong DST, Wang YJ, et al. Chemical and biological approaches synergize to ameliorate protein-folding diseases. Cell 2008;134:769–81. 18775310 10.1016/j.cell.2008.06.037 PMC2650088

[8] Powers ET, Morimoto RI, Dillin A, et al. Biological and chemical approaches to diseases of proteostasis deficiency. Annu Rev Biochem 2009;78:959–91. 19298183 10.1146/annurev.biochem.052308.114844

[9] Ong DST, Kelly JW. Chemical and/or biological therapeutic strategies to ameliorate protein misfolding diseases. Curr Opin Cell Biol 2011;23:231–8. 21146391 10.1016/j.ceb.2010.11.002 PMC3078197

[10] Wang F, Song WS, Brancati G, Segatori L. Inhibition of endoplasmic reticulum-associated degradation rescues native folding in loss of function protein misfolding diseases. J Biol Chem 2011;286:43454–64. 22006919 10.1074/jbc.M111.274332 PMC3234808

[11] Kishnani PS, Corzo D, Nicolino M, et al. Recombinant human acid [alpha]-glucosidase: major clinical benefits in infantile-onset Pompe disease. Neurology 2007;68:99–109. 17151339 10.1212/01.wnl.0000251268.41188.04

[12] Strothotte S, Strigl-Pill N, Grunert B, et al. Enzyme replacement therapy with alglucosidase alfa in 44 patients with late-onset glycogen storage disease type 2: 12-month results of an observational clinical trial. J Neurol 2010;257:91–7. 19649685 10.1007/s00415-009-5275-3

[13] van der Ploeg AT, Clemens PR, Corzo D, et al. A randomized study of alglucosidase alfa in late-onset pompe's disease. N Engl J Med 2010;362:1396–406. 20393176 10.1056/NEJMoa0909859

[14] Harlaar L, Hogrel JY, Perniconi B, et al. Large variation in effects during 10 years of enzyme therapy in adults with Pompe disease. Neurology 2019;93:e1756–67. 31619483 10.1212/WNL.0000000000008441 PMC6946483

[15] Chien YH, Lee NC, Thurberg BL, et al. Pompe disease in infants: improving the prognosis by newborn screening and early treatment. Pediatrics 2009;124:e1116–25. 19948615 10.1542/peds.2008-3667

[16] Kishnani PS, Corzo D, Leslie ND, et al. Early treatment with alglucosidase alfa prolongs long-term survival of infants with Pompe disease. Pediatric Res 2009;66:329–35. 10.1203/PDR.0b013e3181b24e94 PMC3129995 19542901

[17] Kishnani PS, Goldenberg PC, DeArmey SL, et al. Cross-reactive immunologic material status affects treatment outcomes in Pompe disease infants. Mol Genet Metab 2010;99:26–33. 19775921 10.1016/j.ymgme.2009.08.003 PMC3721340

[18] Raben N, Danon M, Gilbert AL, et al. Enzyme replacement therapy in the mouse model of Pompe disease. Mol Genet Metab 2003;80:159–69. 14567965 10.1016/j.ymgme.2003.08.022

[19] Xu YH, Ponce E, Sun Y, et al. Turnover and distribution of intravenously administered mannose-terminated human acid beta-glucosidase in murine and human tissues. Pediatr Res 1996;39:313–22. 8825806 10.1203/00006450-199602000-00021

[20] Shen JS, Edwards NJ, Bin Hong Y, Murray GJ. Isofagomine increases lysosomal delivery of exogenous glucocerebrosidase. Biochem Biophys Res Commun 2008;369:1071–5. 18328804 10.1016/j.bbrc.2008.02.125 PMC2374924

[21] Benjamin ER, Khanna R, Schilling A, et al. Co-administration with the pharmacological chaperone at1001 increases recombinant human α-galactosidase A tissue uptake and improves substrate reduction in Fabry mice. Mol Ther 2012;20:717–26. 22215019 10.1038/mt.2011.271 PMC3321591

[22] Wenk J, Hille A, von Figura K. Quantitation of Mr-46000 and Mr-300000 mannose-6-phosphate receptors in human-cells and tissues. Biochem Int 1991;23:723–32. 1651728

[23] Koeberl DD, Luo XY, Sun BD, et al. Enhanced efficacy of enzyme replacement therapy in Pompe disease through mannose-6-phosphate receptor expression in skeletal muscle. Mol Genet Metab 2011;103:107–12. 21397538 10.1016/j.ymgme.2011.02.006 PMC3101281

[24] Fukuda T, Ahearn M, Roberts A, et al. Autophagy and mistargeting of therapeutic enzyme in skeletal muscle in Pompe disease. Mol Ther 2006;14:831–9. 17008131 10.1016/j.ymthe.2006.08.009 PMC2693339

[25] Fukuda T, Ewan L, Bauer M, et al. Dysfunction of endocytic and autophagic pathways in a lysosomal storage disease. Ann Neurol 2006;59:700–8. 16532490 10.1002/ana.20807

[26] Raben N, Baum R, Schreiner C, et al. When more is less: excess and deficiency of autophagy coexist in skeletal muscle in Pompe disease. Autophagy 2009;5:111–3. 19001870 10.4161/auto.5.1.7293 PMC3257549

[27] Fan JQ. A counterintuitive approach to treat enzyme deficiencies: use of enzyme inhibitors for restoring mutant enzyme activity. Biol Chem 2008;389:1–11. 18095864 10.1515/BC.2008.009

[28] Parenti G. Treating lysosomal storage diseases with pharmacological chaperones: from concept to clinics. EMBO Mol Med 2009;1:268–79. 20049730 10.1002/emmm.200900036 PMC3378140

[29] Gomes CM. Protein misfolding in disease and small molecule therapies. Curr Top Med Chem 2012;12:2460–9. 23339300 10.2174/1568026611212220002

[30] Parenti G, Andria G, Valenzano KJ. Pharmacological chaperone therapy: preclinical development, clinical translation, and prospects for the treatment of lysosomal storage disorders. Mol Ther 2015;23:1138–48. 25881001 10.1038/mt.2015.62 PMC4817787

[31] Porto C, Cardone M, Fontana F, et al. The pharmacological chaperone N-butyldeoxynojirimycin enhances enzyme replacement therapy in Pompe disease fibroblasts. Mol Ther 2009;17:964–71. 19293774 10.1038/mt.2009.53 PMC2835191

[32] Porto C, Pisani A, Rosa M, et al. Synergy between the pharmacological chaperone 1-deoxygalactonojirimycin and the human recombinant alpha-galactosidase A in cultured fibroblasts from patients with Fabry disease. J Inherit Metab Dis 2012;35:513–20. 22187137 10.1007/s10545-011-9424-3

[33] Valenzano KJ, Khanna R, Powe AC, et al. Identification and characterization of pharmacological chaperones to correct enzyme deficiencies in lysosomal storage disorders. Assay Drug Dev Technol 2011;9:213–35. 21612550 10.1089/adt.2011.0370 PMC3102255

[34] Bellomo F, Medina DL, De Leo E, et al. High-content drug screening for rare diseases. J Inherit Metab Dis 2017;40:601–7. 28593466 10.1007/s10545-017-0055-1

[35] Motabar O, Shi ZD, Goldin E, et al. A new resorufin-based alpha-glucosidase assay for high-throughput screening. Anal Biochem 2009;390:79–84. 19371716 10.1016/j.ab.2009.04.010 PMC2737366

[36] Zheng W, Padia J, Urban DJ, et al. Three classes of glucocerebrosidase inhibitors identified by quantitative high-throughput screening are chaperone leads for Gaucher disease. Proc Natl Acad Sci USA 2007;104:13192–7. 17670938 10.1073/pnas.0705637104 PMC1936979

[37] Urban DJ, Zheng W, Goker-Alpan O, et al. Optimization and validation of two miniaturized glucocerebrosidase enzyme assays for high throughput screening. Comb Chem High Throughput Screen 2008;11:817–24. 19075603 10.2174/138620708786734244 PMC2668958

[38] Joosten A, Decroocq C, de Sousa J, et al. A systematic investigation of iminosugar click clusters as pharmacological chaperones for the treatment of Gaucher disease. ChemBioChem 2014;15:309–19. 24375964 10.1002/cbic.201300442

[39] Tropak MB, Blanchard JE, Withers SG, et al. High-throughput screening for human lysosomal beta-N-acetyl hexosaminidase inhibitors acting as pharmacological chaperones. Chem Biol 2007;14:153–64. 17317569 10.1016/j.chembiol.2006.12.006 PMC1989145

[40] Bruckmann C, Repo H, Kuokkanen E, et al. Systematic structure-activity study on potential chaperone lead compounds for acid α-glucosidase. ChemMedChem 2012;7:1943–53. 22969039 10.1002/cmdc.201200309

[41] Porto C, Ferrara MC, Meli M, et al. Pharmacological enhancement of alpha-glucosidase by the allosteric chaperone n-acetylcysteine. Mol Ther 2012;20:2201–11. 22990675 10.1038/mt.2012.152 PMC3519985

[42] Parenti G, Porto C, Moracci M, et al. New allosteric non-inhibitory chaperone of the lysosomal acid alpha-glucosidase, useful for treating pathological condition including lysosomal storage disease, which is Pompe disease, and cystinosis, Danon disease and Fabry disease. Patent application number WO2013182652-A1; 2013. p. 2858638-A1.

[43] Roig-Zamboni V, Cobucci-Ponzano B, Iacono R, et al. Structure of human lysosomal acid α-glucosidase–a guide for the treatment of Pompe disease. Nat Commun 2017;8:1111. 29061980 10.1038/s41467-017-01263-3 PMC5653652

[44] Niesen FH, Berglund H, Vedadi M. The use of differential scanning fluorimetry to detect ligand interactions that promote protein stability. Nat Protoc 2007;2:2212–21. 17853878 10.1038/nprot.2007.321

[45] Vivoli M, Novak HR, Littlechild JA, Harmer NJ. Determination of protein-ligand interactions using differential scanning fluorimetry. J Vis Exp 2014;91:51809. 10.3791/51809 PMC4692391 25285605

[46] Rebouche CJ. Kinetics, pharmacokinetics, and regulation of L-carnitine and acetyl-L-carnitine metabolism. Ann N Y Acad Sci 2004;1033:30–41. 15591001 10.1196/annals.1320.003

[47] Pekala J, Patkowska-Sokola B, Bodkowski R, et al. L-carnitine-metabolic functions and meaning in humans life. Curr Drug Metab 2011;12:667–78. 21561431 10.2174/138920011796504536

[48] Rebouche CJ. Effect of dietary carnitine isomers and gamma-butyrobetaine on L-carnitine biosynthesis and metabolism in the rat. J Nutr 1983;113:1906–13. 6619971 10.1093/jn/113.10.1906

[49] Eknoyan G, Latos DL, Lindberg J, National Kidney Foundation Carnitine Consensus Conference. Practice recommendations for the use of L-carnitine in dialysis-related carnitine disorder. National Kidney Foundation Carnitine Consensus Conference. Am J Kidney Dis 2003;41:868–76. 12666074 10.1016/s0272-6386(03)00110-0

[50] Li JM, Li LY, Zhang YX, et al. Functional differences between L- and D-carnitine in metabolic regulation evaluated using a low-carnitine Nile tilapia model. Br J Nutr 2019;122:625–38. 32124711 10.1017/S000711451900148X

[51] Lieberman RL, Wustman BA, Huertas P, et al. Structure of acid beta-glucosidase with pharmacological chaperone provides insight into Gaucher disease. Nat Chem Biol 2007;3:101–7. 17187079 10.1038/nchembio850

[52] Parenti G, Zuppaldi A, Gabriela Pittis M, et al. Pharmacological enhancement of mutated α-glucosidase activity in fibroblasts from patients with Pompe disease. Mol Ther 2007;15:508–14. 10.1038/sj.mt.6300074 28182897

[53] Landon MR, Lieberman RL, Hoang QQ, et al. Detection of ligand binding hot spots on protein surfaces via fragment-based methods: application to DJ-1 and glucocerebrosidase. J Comput Aided Mol Des 2009;23:491–500. 19521672 10.1007/s10822-009-9283-2 PMC2889209

[54] Patnaik S, Zheng W, Choi JH, et al. Discovery, structure-activity relationship, and biological evaluation of noninhibitory small molecule chaperones of glucocerebrosidase. J Med Chem 2012;55:5734–48. 22646221 10.1021/jm300063b PMC3400126

[55] Marugan JJ, Zheng W, Motabar O, et al. Evaluation of 2-thioxo-2,3,5,6,7,8-hexahydropyrimido[4,5-d]pyrimidin-4(1H)-one analogues as GAA activators. Eur J Med Chem 2010;45:1880–97. 20206419 10.1016/j.ejmech.2010.01.027 PMC2892120

[56] Flanagan JJ, Rossi B, Tang K, et al. The pharmacological chaperone 1-deoxynojirimycin increases the activity and lysosomal trafficking of multiple mutant forms of acid alpha-glucosidase. Hum Mutat 2009;30:1683–92. 19862843 10.1002/humu.21121

[57] Martins LO, Santos H. Accumulation of mannosylglycerate and di-myo-inositol-phosphate by pyrococcus furiosus in response to salinity and temperature. Appl Environ Microbiol 1995;61:3299–303. 16535119 10.1128/aem.61.9.3299-3303.1995 PMC1388573

[58] Jorge CD, Borges N, Santos H. A novel pathway for the synthesis of inositol phospholipids uses cytidine diphosphate (CDP)-inositol as donor of the polar head group. Environ Microbiol 2015;17:2492–504. 25472423 10.1111/1462-2920.12734

[59] Mattos C, Bellamacina CR, Peisach E, et al. Multiple solvent crystal structures: probing binding sites, plasticity and hydration. J Mol Biol 2006;357:1471–82. 16488429 10.1016/j.jmb.2006.01.039

[60] Buhrman G, O'Connor C, Zerbe B, et al. Analysis of binding site hot spots on the surface of Ras GTPase. J Mol Biol 2011;413:773–89. 21945529 10.1016/j.jmb.2011.09.011 PMC3247908

[61] Sabanes Zariquiey F, de Souza JV, Bronowska AK. Cosolvent analysis toolkit (CAT): a robust hotspot identification platform for cosolvent simulations of proteins to expand the druggable proteome. Sci Rep 2019;9:19118. 31836830 10.1038/s41598-019-55394-2 PMC6910964

[62] Fuglestad B, Kerstetter NE, Wand AJ. Site-resolved and quantitative characterization of very weak protein-ligand interactions. ACS Chem Biol 2019;14:1398–402. 31246002 10.1021/acschembio.9b00353 PMC7051831

[63] Cavazza C, Use of acetyl D-carnitine in the therapeutic treatment of glaucoma, and pharmaceutical compositions useful in such treatment. 5,432,199, 11/07/1995; 1995.

[64] Pepine CJ, Welsch MA, Therapeutic potential of L-carnitine in patients with angina pectoris. Vol. 162. Dordrecht: Springer; 1995.

