# Supplementary material for: Carnitine is a pharmacological allosteric chaperone of the human lysosomal α-glucosidase
Source: J Enzyme Inhib Med Chem. 2021 Sep 27;36(1):2068–79. doi: 10.1080/14756366.2021.1975694 (PMC8477953; doi:10.1080/14756366.2021.1975694)
Supplement: Supplemental Material [file IENZ_A_1975694_SM4378.zip › IENZ_A_1975694_SuppFigure.pdf]

Time course of the effect of L-, and D-CAR on rhGAA

a

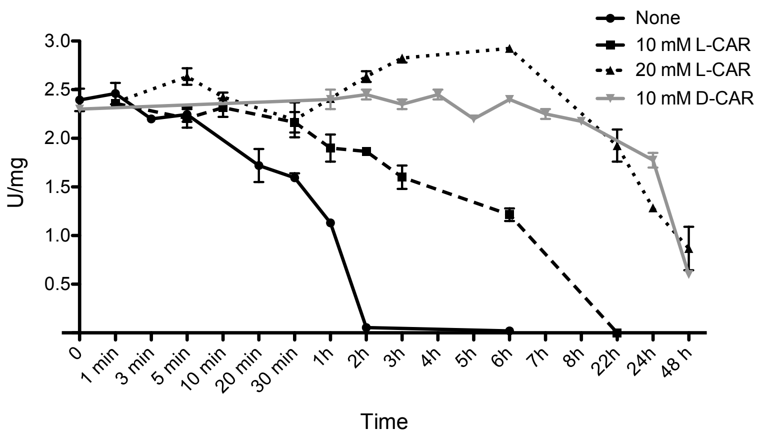

Effect of L-CAR on the specific activity of rhGAA

b

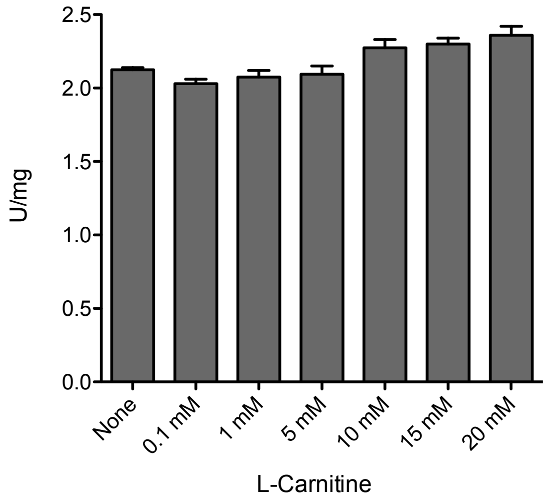

Figure S1

Effect of D-CAR and A-D-CAR on the activity and stability of rhGAA

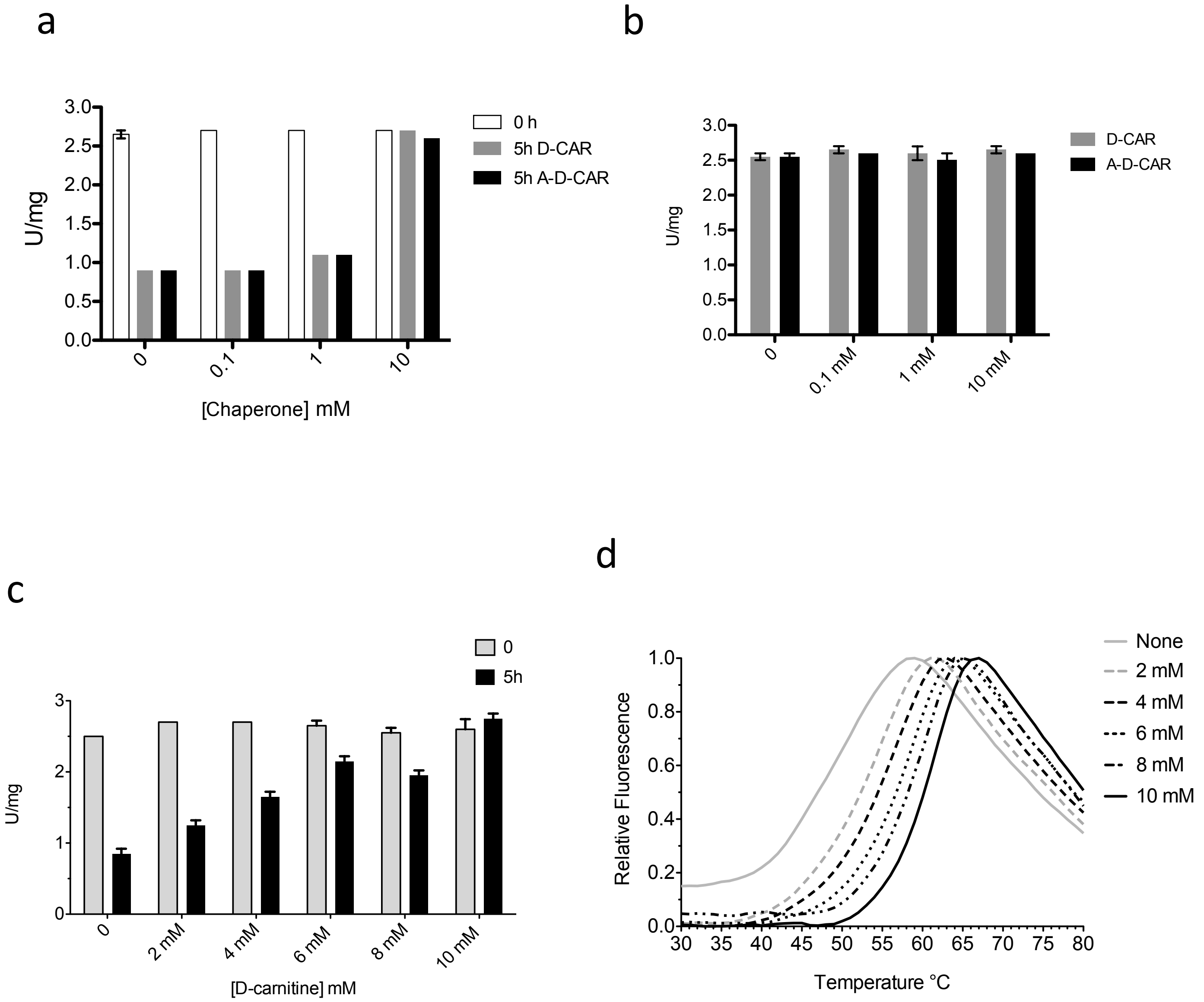

e

	None	2 mM	4 mM	6 mM	8 mM	10 mM
**Tm [° C]**	48.4 ± 0.2	52.3 ± 0.1	54.4 ± 0.1	56.8 ± 0.2	58.5 ± 0.3	60.1 ± 0.2
**ΔTm [° C]**		+3.9 ± 0.1	+6.0 ± 0.1	+8.4 ± 0.2	+10.1 ± 0.2	+11.7 ± 0.2

Figure S2

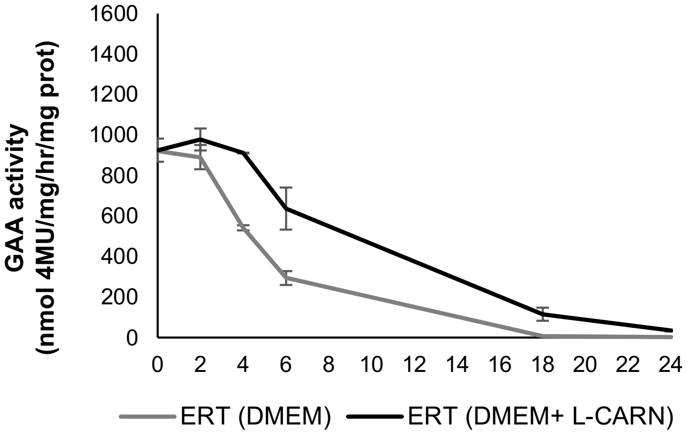
GAA activity
(nmol 4MU/mg/hr/mg prot)
1600
1400
1200
1000
800
600
400
200
0
0
2
4
6
8
10
12
14
16
18
20
22
24
ERT (DMEM)
ERT (DMEM+ L-CARN)

Figure S3
