# Supplementary material for: Carnitine is a pharmacological allosteric chaperone of the human lysosomal α-glucosidase
Source: J Enzyme Inhib Med Chem. 2021 Sep 27;36(1):2068–79. doi: 10.1080/14756366.2021.1975694 (PMC8477953; doi:10.1080/14756366.2021.1975694)
Supplement: Supplemental Material [file IENZ_A_1975694_SM4378.zip › IENZ_A_1975694_SuppText.pdf]

SUPPORTING MATERIAL

Carnitine is a Pharmacological Allosteric Chaperone of the Human Lysosomal α-Glucosidase

Roberta Iacono[1, 2¶], Nadia Minopoli[3, ¶], Maria Carmina Ferrara[2], Antonietta Tarallo[3], Carla Damiano[3], Caterina Porto[3], Sandra Strollo[3], Véronique Roig-Zamboni[4], Gianfranco Peluso[6], Gerlind Sulzenbacher[4], Beatrice Cobucci-Ponzano[2], Giancarlo Parenti[3,5], Marco Moracci[1, 2, *]

[1]Department of Biology, University of Naples "Federico II", Complesso Universitario di Monte S. Angelo, Via Cupa Nuova Cinthia 21, 80126 Naples, Italy

[2]Institute of Biosciences and Bioresources – CNR, Via P. Castellino 111, 80131, Naples, Italy

[3]Telethon Institute of Genetics & Medicine, Via Campi Flegrei 34, 80078, Pozzuoli, Italy

[4]Centre National de la Recherche Scientifique (CNRS), Aix-Marseille Univ, AFMB, 163 Avenue de Luminy, 13288 Marseille, France

[5]Department of Translational Medical Sciences, Federico II University, Via S. Pansini 5, 80131, Naples, Italy

[6]Research Institute on Terrestrial Ecosystems, UOS Naples-CNR, Via P. Castellino 111, 80131, Naples, Italy

[¶]These authors contributed equally to the work

*Author to whom correspondence should be addressed: Dr. Marco Moracci. Tel +39-081-679046; Fax +39-081-679233; e-mail: marco.moracci@unina.it

Figure captions

Figure S1. ***Effect of L-CAR and D-CAR on rhGAA***

a) Time course of the stabilizing effect of L- and D-CAR on the activity of rhGAA. The stability of rhGAA activity was measured in the absence and in the presence of L-CAR (10 or 20 mM) and D-CAR for 48 h. *b) Effect of L-CAR on rhGAA activity*. The specific activity of rhGAA was measured in the absence and in the presence of L-CAR at various concentrations.

Figure S2. ***Comparison of the effect of D-CAR and A-D-CAR on the stability of rhGAA*** *(a) Effect on the rhGAA stability*. The specific activity of rhGAA was measured in the absence and in the presence of D- and A-D-CAR at various concentrations (0.1-10 mM). *(b) Effect on the rhGAA activity:* D- and A-D-CAR at various concentrations (0.1-10 mM) were incubated with rhGAA and the enzymatic activity was measured after 5 h of incubation at pH 7.4. *(c) Effect of D-CAR on the stability of the rhGAA activity*. rhGAA was incubated alone or with D-CAR (2-10 mM) in sodium phosphate buffer pH 7.4 at 37 °C. After 5h, the residual α-glucosidase activity was measured with the standard assay. *(d) Effect of D-CAR on the structural stability of rhGAA:* D-CAR was incubated with rhGAA at 5 concentrations (from 2 to 10 mM). Changes in the fluorescence of SYPRO Orange were monitored by DSF as a function of temperature at pH 7.4. *(e) Summary of the T_ms measured by DSF:* T_m values were calculated according to Niesen et al., 2007 [1]. The standard deviations for each melting temperature were calculated from three replicates.

Figure S3. ***Effect of L-CAR on rhGAA stability in the medium***

PD fibroblasts were incubated in Dulbecco's modified Eagle's medium (DMEM) in the presence or in the absence of L-CAR 10 mM (black and grey lines, respectively). GAA activity decreased over time, with significant differences between rhGAA in combination with L-CAR and rhGAA alone already detectable after 2 hrs.

1. Niesen, F. H.; Berglund, H.; Vedadi, M. The use of differential scanning fluorimetry to detect ligand interactions that promote protein stability. *Nature Protocols* **2007,** 2, 2212-2221.
